# Commentary: Special Report: The Biology of Inequalities in Health: The Lifepath Consortium

**DOI:** 10.3389/fpubh.2020.504530

**Published:** 2020-10-29

**Authors:** Virginia Ghiara

**Affiliations:** Department of Philosophy, University of Kent, Canterbury, United Kingdom

**Keywords:** lifepath consortium, social determinants of health, causality, health inequalities, philosophy of science

In the health and social sciences, there is a general agreement that health is more than a biological phenomenon (1–3), yet the causal relationships between the social and the biological dimensions of health have long remained elusive. This commentary aims at shedding light on how the project *Lifepath* has contributed to improving our understanding of such interactions between “the social” and “the biological.”. *Lifepath* is a research project funded by the European Commission Horizon 2020 programme aimed at investigating the effects of socioeconomic inequalities on the biology of healthy aging. *Lifepath*'s main research questions include how inequalities impact on health, what are the biological mechanisms underlying social differences in healthy aging, and what policies can reduce social differences and promote healthy aging. When I worked as a visiting researcher at the School of Public Health of Imperial College London, I had the opportunity to discuss the assumptions and methods underlying this project with the Principal Investigator, Paolo Vineis, and some *Lifepath* researchers, and to observe some processes by which they got to their scientific findings.

Recently, the *Lifepath* consortium has published a Special Report (4) summarizing the main results of *Lifepath*. In this commentary, I will argue that the approach used in *Lifepath* not only helps to overcome the fragmentation of concepts that generally characterizes the health and epidemiological studies, but paves also the way for new causal discussions on the complexity of health.

## Lifepath's Life-Course Approach

While the causal impact of socio-economic factors on health has been long studied (5–7), the current models of health and illness are still dominated by a clear-cut distinction between socioeconomic and biological factors. Socio-economic factors are treated as distal causes that are far from the health effects under consideration, biological factors are proximal, located within the body and directly influencing health and disease (8, 9). This distinction can be partly explained considering the challenges of integrating such dimensions. One the one hand, the biological trajectory from exposure to disease development can be now studied through biomarkers, which allow researchers to identify some key biological components of the mechanisms of health and disease. On the other hand, concepts such as socio-economic position appear to be intangible social constructs that slip right out of our grasp.

The project *Lifepath*, through a life-course approach, has taken a holistic view of health to bridge the gap between the biological and social dimensions. Among the methodological and conceptual novelties developed in *Lifepath*, one in particular stands up: the attempt to capture in a systematic way the biological embodiment of social adversity by means of concepts such as allostatic load, accelerated aging and inflammation (4). *Lifepath*'s discussions and studies of such concepts have contributed, from a methodological point of view, to filling the gap in our understanding of how socio-economic exposure is translated into precise biological alterations. Moreover, they have also provided good conceptual foundation for a new model where “the social” and “the biological” dynamically interact, and the biological factors are embedded in organisms which, in turn, are embedded in societies.

## The Need For A New Causal Account

The process of overcoming a clear-cut distinction between “the social” and “the biological” inevitably requires a new causal account where both social and biological factors can play an active causal role in ill-health or disease. While the traditional causal accounts, based on biological entities and activities, are difficult to adapt to such interactions, a new view of causation, based on the concept of processes, has recently emerged to accommodate the life-course approach (10–12). This new account recognizes biological markers as “picking up signals” that do not necessarily correspond to “objects out there” and interprets the new biomarker studies as reconstructions of the continuum from macro-level exposures to micro-level changes. *Lifepath* studies are in line with this approach: they have used biomarkers to follow longitudinal threads of marks from socio-economic exposures to biological alterations. An illustrating example is the way in which *Lifepath* studies have used the concept of allostatic load, a composite score developed to measure how much it costs the organism to adapt to the stressful conditions encountered in life. Allostatic load biomarkers were used as “picking up signals” to explore the causal process linking social experiences and later-in-life physiological conditions (4). *Lifepath* studies based on concepts such as inflammation, allostatic load and epigenetic clock used biomarkers as “signal” to reconstruct the biological imprinting process (embodiment) of social variables, however the underlying causal account has not been directly investigated within the project. A clear stance, nevertheless, would help to clarify *Lifepath* conceptual and methodological approach and, notably, would also allow *Lifepath* to distance itself from reductionist positions, whereby the “social” ends up being understood as “the cause of the (biological) causes.”

## Exploring The Social

One of the most important messages of *Lifepath* is that the effect of socio-economic position on health is not fully driven by established health risk factors such as smoking and drinking. Although *Lifepath* studies have confirmed the importance of behaviors in health, they have also provided new evidence showing that the social dimension cannot be reduced to a set of risky behaviors, and that further social factors including family economic pressure, harsh parenting and early life adversity can act as key drivers of later-in-life health conditions. This consideration has two important implications. First, it brings to the fore the fact that “the social” is a heterogeneous dimension including more than individual activities: its components are social, psychological, cultural, and behavioral factors. Second, it helps to recognize the inadequacy of the assumption that the causes and treatments of disease should be located solely within the individual. The former implication has been partially investigated in *Lifepath*, but further efforts are needed to develop a comprehensive understanding of the various components. Integrating *Lifepath* findings with sociological and psychological discussions is not a trivial task and requires more than fitting together fixed pieces of “knowledge” taken from different disciplines. Concepts, methods and approaches usually require researchers to share epistemic principles and tacit knowledge. Yet, this challenge is as important as ever to disambiguate key mechanistic factors explored in *Lifepath* such as stress and adverse experiences, which cannot be adequately conceptualized within any singular discipline (13–15).

The recognition that the causes and treatments of disease should not be located solely within the individual has led *Lifepath* to focus on a broad group of policies. *Lifepath* has distanced itself from the simplified approaches that interpret health problems in terms of poor lifestyle choices, and has acknowledged the importance of tackling the determinants of health at several levels. This consideration is combined with the recognition that socioeconomic inequalities influence several stages of the disease continuum, with the consequence that health policies can be developed both to prevent health effects and to reverse them. *Lifepath* studies have emphasized the importance of the early years of life and provided support to a growing body of literature advocating for preventive interventions (16–18). Preventive interventions can be delivered at the individual level (to target behaviors and attitudes), at the community level (to encourage and promote changes in civil society), and at the structural level (to address socio-economic inequalities). Identifying what interventions (or packages of interventions) are successful in preventing health inequalities is challenging, especially considering that different interventions can interact not only with each other, but also with the environment. The cultural context, hence, needs to be taken into account to assess the external validity of a policy intervention (i.e., to answer the question whether a particular intervention would achieve similar results in different populations) (19). The mixed picture obtained in *Lifepath* from the experimental and quasi-experimental studies examining the effects of conditional cash transfers and compulsory schooling laws on physical health and psychological well-being clearly reflects these challenges, and sheds light on the importance of building a new model that reflects the dynamic complexity of the determinants of health.

## Author Contributions

The author confirms being the sole contributor of this work and approved it for publication.

## Conflict of Interest

The author declares that the research was conducted in the absence of any commercial or financial relationships that could be construed as a potential conflict of interest.
